# SecretEPDB: a comprehensive web-based resource for secreted effector proteins of the bacterial types III, IV and VI secretion systems

**DOI:** 10.1038/srep41031

**Published:** 2017-01-23

**Authors:** Yi An, Jiawei Wang, Chen Li, Jerico Revote, Yang Zhang, Thomas Naderer, Morihiro Hayashida, Tatsuya Akutsu, Geoffrey I. Webb, Trevor Lithgow, Jiangning Song

**Affiliations:** 1 College of Information Engineering, Northwest A&F University, Yangling 712100, China; 2 Monash Centre for Data Science, Faculty of Information Technology, Monash University, Melbourne, VIC 3800, Australia; 3 School of Electronic and Computer Engineering, Peking University, Beijing 100871, China; 4 Infection and Immunity Program, Biomedicine Discovery Institute and Department of Microbiology, Monash University, Melbourne, VIC 3800, Australia; 5 Monash Bioinformatics Platform, Monash University, Melbourne, VIC 3800, Australia; 6 Infection and Immunity Program, Biomedicine Discovery Institute and Department of Biochemistry and Molecular Biology, Monash University, Melbourne, VIC 3800, Australia; 7 Bioinformatics Center, Institute for Chemical Research, Kyoto University, Uji, Kyoto 611-0011, Japan

## Abstract

Bacteria translocate effector molecules to host cells through highly evolved secretion systems. By definition, the function of these effector proteins is to manipulate host cell biology and the sequence, structural and functional annotations of these effector proteins will provide a better understanding of how bacterial secretion systems promote bacterial survival and virulence. Here we developed a knowledgebase, termed SecretEPDB (Bacterial Secreted Effector Protein DataBase), for effector proteins of type III secretion system (T3SS), type IV secretion system (T4SS) and type VI secretion system (T6SS). SecretEPDB provides enriched annotations of the aforementioned three classes of effector proteins by manually extracting and integrating structural and functional information from currently available databases and the literature. The database is conservative and strictly curated to ensure that every effector protein entry is supported by experimental evidence that demonstrates it is secreted by a T3SS, T4SS or T6SS. The annotations of effector proteins documented in SecretEPDB are provided in terms of protein characteristics, protein function, protein secondary structure, Pfam domains, metabolic pathway and evolutionary details. It is our hope that this integrated knowledgebase will serve as a useful resource for biological investigation and the generation of new hypotheses for research efforts aimed at bacterial secretion systems.

In the course of pathogenesis, bacteria utilize highly evolved secretion systems to translocate (secrete) proteins into host cells. A majority of these secreted proteins are enzymes, toxins or “effectors”; with effector proteins functioning to subvert the pathways of host cells to facilitate bacterial pathogenicity^1^,^2^. A growing number of bacterial secretion systems have been identified to date, from type I to type IX^2^,^3^,^4^,^5^,^6^,^7^. They play important roles in mediating the interactions of bacteria with their host cells, and thus determine infection outcomes^8^. For example, bacteria are able to degrade the extracellular matrix and cell walls of host niches using secreted enzymes^3^,^9^. These enzymes are exported to the environment and their secretion is mainly through the secretion systems of type I (T1SS), type II (T2SS) or type V (T5SS)^10^.

Effector proteins are translocated into host cells predominantly by the type III secretion system (T3SS), type IV secretion system (T4SS) or type VI secretion system (T6SS)^1^,^11^,^12^,^13^. Of these, the T3SS has been most extensively studied both structurally and functionally and has been shown to exist in diverse bacterial species^6^,^7^. Both animals and plants can be infected by pathogens that use T3SS effectors (T3SEs)^3^,^6^,^7^,^14^. The T4SS is regarded as one of the most functionally diverse bacterial secretion system, both in terms of transported substrates and targeted recipients^15^. The T4SS is characterized as a large family of macromolecule transporter systems that incorporates three recognized sub-families: bona fide effector protein transport systems (e.g. Dot/Icm from *Legionella*; CagPAI from *Helicobacter*), machinery for DNA uptake/release (e.g. Tra from *Neisseria*) and conjugation systems for the transfer of genetic material between bacteria as well as from bacteria to eukaryotic cells (e.g. VirB and Trw from *Bartonella*)^16^,^17^. Only more recently discovered, necessarily less is known about the T6SS which is composed of a contractile, needle-tube puncturing apparatus to deliver effectors into host or other bacterial cells^13^.

Through secretion of effector proteins and interaction with host factors, protein secretion represents an important aspect of bacterial physiology, and a crucial means for adaptation and survival within host niches. With the functional importance of bacteria secretion systems in mediating the mutualistic symbiotic or pathogenic relationships^18^, experimental and computational studies have been aimed at understanding the role of effector proteins in host-pathogen interactions^19^,^20^,^21^,^22^,^23^,^24^,^25^,^26^,^27^,^28^,^29^,^30^. Web servers for predicting T3SS, T4SS or T6SS effector proteins from genome sequence data have been established^31^,^32^,^33^,^34^. However, to the best of our knowledge, there are currently no available knowledge-bases or resources that document and curate the annotations for effector proteins of the T3SS, T4SS and T6SS. Considering the importance of bacteria secretion systems, comprehensive sequence, structural and functional annotations of their effector proteins will provide a better understanding of their importance.

To bridge this knowledge gap, we developed a new web-based resource termed SecretEPDB (Bacterial Secreted Effector Protein DataBase), for a comprehensive annotation of effector proteins secreted by the bacterial T3SS, T4SS and T6SS. SecretEPDB provides detailed annotations for the three types of effector proteins, through manual extraction and integration using currently available databases or the literature from PubMed. Importantly, the database has been strictly curated to ensure that all effector protein entries in SecretEPDB are supported by experimental evidence published in the scientific literature of being secreted by T3SS, T4SS or T6SS. In addition, several key features of the developed SecretEPDB are as follows:

1. Protein 3D structural information is available in SecretEPDB. For each entry with available structural information, the corresponding Protein Data Bank (PDB)^35^ accession numbers, experimental structural determination methods, and the 3D structures were extracted and made available.
2. For the entries with UniProt (http://www.uniprot.org/)^36^ accession numbers, their functional sites and domains were assembled and can be visualized with the IBS (Illustrator of Biological Sequences) program^37^ to provide an enhanced visualization of the sequence context information.
3. Data visualization is also available for multiple sequence alignment (MSA) of each entry with Alignment-to-Html^38^, a third-party JavaScript tool. Alignment-to-Html enables SecretEPDB to visualize MSAs with overlapped functional domains and/or sites. In addition, SecretEPDB allows users to search protein motifs with a user-friendly interface and an option of exporting multiple retrieved sequences in the FASTA format as plain text or directly to MS Word.
4. SecretEPDB provides annotations of metabolic/signaling pathway for each entry by cross-referencing the KEGG database^39^ where such information is available. Pathway annotations are important for understanding the functional roles of the effector proteins within the host cells.
5. SecretEPDB includes single point mutations and their pathogenicity annotations of each protein entry. For each mutation, detailed annotations including disease type and the corresponding reference papers are provided.
6. Post-translational modification sites are crucial for protein function. SecretEPDB includes kinase-specific phosphorylation site annotations predicted by the Group-based Prediction System (GPS) program^40^, which was employed to provide the annotations of predicted kinase-specific phosphorylation sites in hierarchy. Identifying phosphorylation sites in partner with their cognate protein kinases provides important information for understanding a variety of related cellular processes that are potentially associated with the effector proteins.
7. In an effort to keep up with the rapid accumulation of experimental data, SecretEPDB allows researchers to submit their most-recent experimental findings of novel effector proteins via an online submission webpage. Please refer to ‘Database utility’ for more information.

## Database construction and content

### Data collection

**Figure 1:**
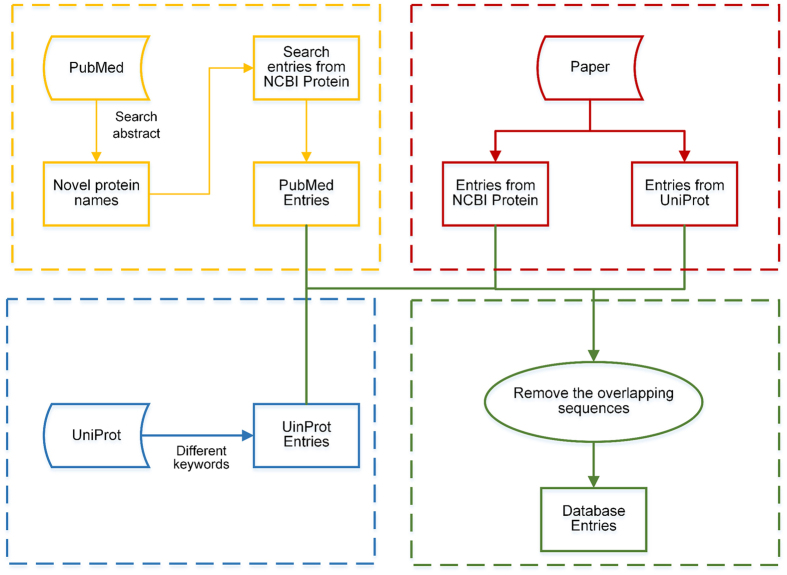
Flowchart of the data collection process in SecretEPDB.

Figure 1 presents the flowchart describing construction of SecretEPDB. Current database entries were extracted from three major resources: UniProt, Datasets from published studies, and the relevant literature (entries were collected from the literature via keyword search in NCBI Protein). A three-step procedure of the entry retrieval and collection is described as follows.

Firstly, keywords including ‘bacterial secretion system’, ‘bacterial secretion effectors’, ‘T3SS’, ‘T4SS’ and ‘T6SS’ were each used to search the entire Swiss-Prot database (i.e. the manually annotated and reviewed dataset of the UniProt database). Expectedly, the search returned a huge number of redundant (and sometimes irrelevant) secreted effector protein candidates. After being carefully reviewed, those proteins that did not belong to any of the three classes (i.e. T3SS, T4SS or T6SS) were disregarded. It is important to note that the obtained entries were required to have accurate and unambiguous descriptions and evidence (such as “secreted by T3SS”, or “translocated into the host cell via the type IV secretion system”). As a result, a list of 169 entries was obtained, including 161 type III secretion system effectors (termed as T3SEs), 4 type IV secretion system effectors (T4SEs) and 4 type VI secretion system effectors (T6SEs).

Secondly, a number of effector proteins were collected from datasets^14^,^20^,^21^,^27^,^28^,^41^ or databases^32^ published in the literature. Note that these proteins were collected from NCBI Protein or UniProt database, which may or may not be in their full-length form. During this step, we extracted complete sequences by searching the accession numbers in NCBI Protein or UniProt database. In addition, a number of entries unavailable for extraction, having been removed from NCBI Protein or UniProt database. After careful curation, we obtained 2538 entries: 1150 T3SEs, 1216 T4SEs and 172 T6SEs. Among these, 1090 T3SEs and 254 T4SEs were derived from the UniProt database (i.e. the TrEMBL database). Table 1 provides lists of the number for each type which were obtained from the various data sources.

Finally, we searched the PubMed abstracts for relevant literature to retrieve experimentally reported T3SS, T4SS and T6SS effector proteins. These entries represent newly discovered effector proteins that may not yet be included in the current databases or datasets. In particular, the abstract of each paper in PubMed was mined using a text-mining technique called Scrapy (http://scrapy.org/), a fast and powerful web crawling tool. We extracted T3SS, T4SS and T6SS proteins and their associated information including their names and accession numbers. The collected information was then used to search against the NCBI protein database to retrieve proteins sequences in the FASTA format. Note, some effectors mentioned in the literature would not be included in SecretEPDB until such time as sequence information is available on these effectors. After this step, a total number of 44 entries, including 27 T3SEs, 8 T4SEs and 9 T6SEs were extracted and added into SecretEPDB.

**Figure 2:**
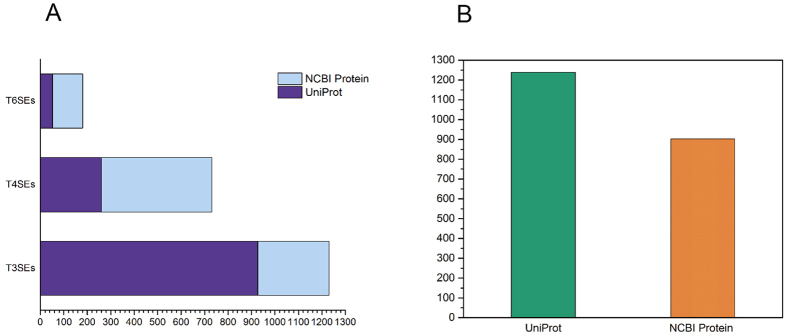
Statistical summary of collected entries currently in SecretEPDB. (**A**) Distribution of effector protein entries according to the original resources used; (**B**) Distribution of entries from UniProt and NCBI protein database.

These steps generated a total of 1338 T3SEs, 1228 T4SEs and 185 T6SEs. We then reduced the sequence redundancy of the collected effector proteins by comparing their UniProt and NCBI Protein accession numbers. Altogether SecretEPDB collected 2142 experimentally verified effectors (1239 entries exist in UniProt and 903 entries exist in the NCBI Protein database). Figure 2 shows the numbers of T3SEs, T4SEs and T6SEs and the distribution of these entries from different resources.

**Figure 3:**
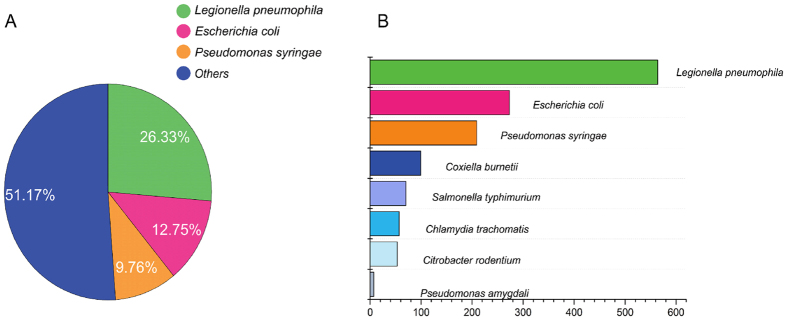
Distribution of collected entries according to bacterial species. (**A**) Distribution of the entries from the three dominant bacterial species; (**B**) Statistical analysis of the entries from the top eight bacterial species.

With the collected effector protein entries in SecretEPDB we conducted a statistical analysis of their distribution across the bacterial species: the most abundant source with 26.33% of entries was *Legionella pneumophila*, followed by *Escherichia coli* (12.75%) and *Pseudomonas syringae* (9.76%). The distribution of collected effector proteins across different species is shown in Fig. 3. These effectors were either published or have been previously used in positive data sets for training machine learning models in past computational studies. This distribution across species is currently biased, which is probably due to two factors. The first one is the biased historical research interest. For example, much of the early work on the T3SS focused on *Salmonella enterica* serovars to establish this organism as the model for T3SE discovery^6^,^46^. The second “bias” derives from the prevalence of effector proteins in some species. For example, recent comprehensive surveys suggest more than three hundred T4SEs are encoded in the genome of some strains of *Legionella pneumophila*^43^,^47^,^48^,^49^.

For all these three secretion systems (T3SS, T4SS and T6SS), the targeting information in the effectors has remained nebulous. Several previous studies using genetics and biochemistry to analyse specific effector proteins of interest suggest that the N- or C-terminal region may carry the targeting information. In the case of *Legionella*, some of the T4SEs depend on a C-terminal targeting signal, a dedicated molecular chaperone (icmS and W), or both in order to be secreted^43^. By way of demonstrating the utility of having a large, experimentally validated set of effector protein sequences to raise hypotheses about T4SS function, the composition and putative preference for conserved amino acids located at the both N- and C-termini of the collected entries was addressed. The collection of sequences offered the opportunity to look for dominant residues in an effector collection (e.g. T4SEs) and within a given species (e.g. the T4SEs in *Legionella*).

**Figure 4:**
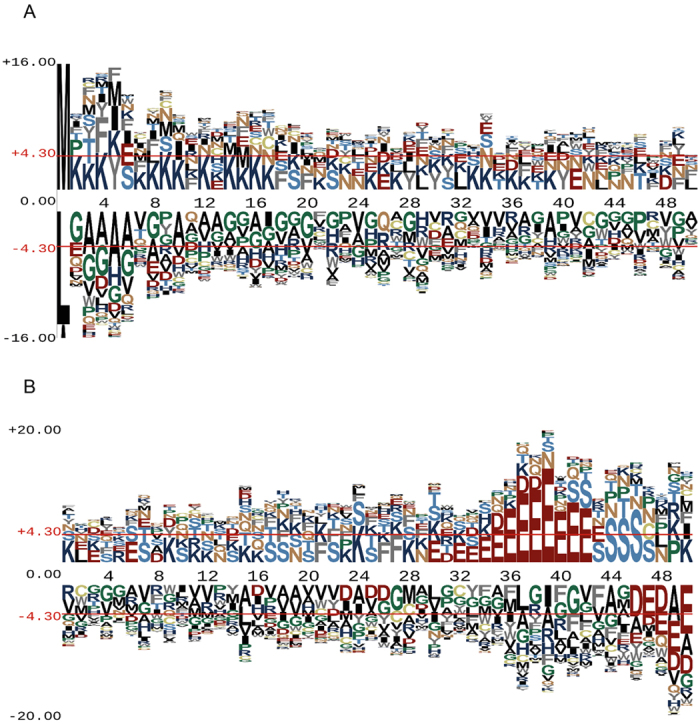
Sequence logos showing the amino acid conservation and preference in T4SEs. Sequence Logo plots of the indicated number of residues in the N-terminal (**A**) and C-terminal (**B**) regions of the collected sequences of T4SEs from *Legionella pneumophila*. The x-axis represents residue numbers, and Amino acids above the *x*-axis are favoured while those underneath the *x*-axis are disfavoured at the corresponding positions. Note that because of the mechanism of protein synthesis, the N-terminal position of a bacterial protein can only ever be methionine (M), isoleucine (I) or leucine (L), with M being vastly the most common.

The background dataset for statistical analysis was based on protein sequences obtained by searching UniProt with “Legionella protein” as the keywords. For each type of effector proteins, the motifs from N- and C-termini were extracted using a window size of 50 amino acids^28^,^29^. The sequence conservation for T4SEs is depicted using pLogo^50^ for the N- terminal motifs and C-terminal motifs of the single dominant species *Legionella* (Fig. 4). Excluding the translation-initiating N-terminal methionine (M) from position 1 (Fig. 4A), two observations become apparent. Firstly, in the case of the C-terminal motifs, there is a striking confirmation of the preponderance of glutamate (E) at positions -9 to -16 for the T4SEs. Furthermore, it becomes clear that there is a strong dis-favoring of glutamate and the other acidic amino acid aspartate (D) from the final five positions at the C-terminus of the sequences (Fig. 4B). These signatures impact on protein translocation^43^,^51^. Secondly, there is a preponderance of lysine (K) residues for 3–4 positions, reoccurring through the N-terminal segment (Fig. 4A). For practical reasons, the alignments are made from position 1, which is an artificial means to register the sequences. Given this, the observed distribution would occur if a periodical presence of lysine e.g. occurring on an aligned face of an alpha-helical segment, were part of a consensus sequence important for recognition and/or translocation. Glycine (G), alanine (A) and proline (P) residues tend to be dis-favored in the N-terminal segments (Fig. 4A), which would be consistent with a helical structure being an important feature of the T4SE. As several effectors rely on the dot/icm chaperones IcmS and W for efficient translocation by T4SS, a reasonable hypothesis would be that these conserved sequence features in a secondary structure context serve as binding sites for chaperones such as IcmS and W. To provide an overview of sequence preferences for N- and C-terminal segment of all the three types of effectors, we also generated sequence logo representations for all the collected entries of T3SS, T4SS and T6SS (Supplementary Figure S1).

### Database contents

For all entries in SecretEPDB, we extracted, manually checked and integrated their annotations from several publicly available databases, including UniProt^36^, NCBI Protein database (http://www.ncbi.nlm.nih.gov/protein), Pfam^52^, KEGG (Kyoto Encyclopedia of Genes and Genomes)^39^ and PDB^53^. From the UniProt database information was extracted forprotein accession number, protein name, bacterial species and functional annotations. We also annotated protein secondary structures, by mapping each entry onto the PDB database using BLAST search. For each structure included in SecretEPDB, an overview snapshot is provided for the structure. In addition to conserved structural elements, protein disordered regions can also be crucial for protein function^54^, with some protein regions being intrinsically disordered and lacking structural information^55^. Given that protein disordered regions may be functionally important^54^,^56^, we used the popular bioinformatics tool VSL2B^57^ to predict natively disordered regions. Where available, disordered region prediction results from the Database of Disordered Protein Prediction (D^2^P^2^)^58^ are provided for protein entries. This provides a general overview of ordered and disordered regions in those proteins. To better display the protein context information, we employed IBS^37^ to present and visualize functional sites and domains in an integrative manner. These functional sites and domains were retrieved using the UniProt accession number for each entry and then used for plotting figures by IBS.

To capture related but non-identical sequence relationships between effectors, both sequence modules and MSAs of each entry were generated and annotated using Strap^59^ in an interactive manner in SecretEPDB. The sequence module provides the amino acid sequence augmented by predicted secondary structure inferred by the SSpro program^60^ included in the SCRATCH suite. The MSAs were generated by Clustal Omega^61^ based on the homologous sequences of each entry, which were retrieved using PSI-BLAST search against the Swiss-Prot database (with an e-value < 0.0001 and sequence identity > 0.8).

The entries in SecretEPDB are deposited in a MySQL relational database. Several website development techniques (including jQuery, Bootstrap, JAVA, Structs and Hibernate) were utilized to implement SecretEPDB, enabling the design of a user-friendly interface, multiple functionalities and enhanced data visualization.

### Database utility

SecretEPDB provides a number of functionalities to optimize the user experience including database searches, browsing, download and new entry submission. A webpage is also available to provide a statistical overview of the current entries in SecretEPDB in terms of the secretion system types, bacterial species (http://secretepdb.erc.monash.edu/statistics.jsp).

**Figure 5:**
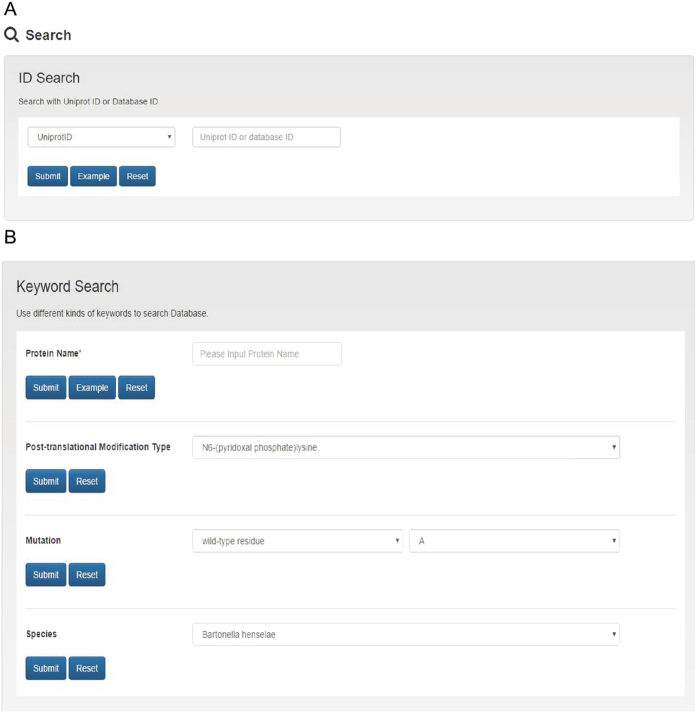
Examples of search options available in SecretEPDB. (**A**) Search option with UniProt ID or SecretEPDB ID; (**B**) Search option with a number of keywords, including protein name, mutation and species.

There are in total currently 2142 proteins in SecretEPDB. The search webpage (http://secretepdb.erc.monash.edu/getDropDownList.action) allows users to search these entries in SecretEPDB in two different ways, i.e. search with the ID and keyword (Fig. 5). For search with the ID, SecretEPDB provides two alternative IDs: UniProt ID and SecretEPDB ID. The former is composed of 6 letters and digits, whereas the latter is drawn from a range of consecutive integers. Searching with the keyword is also straightforward. Several types of keywords are provided including protein name, mutation and bacterial species. For these different search options, a corresponding example is available for guiding users to search the database. By clicking the ‘Example’ button, users can promptly get the example keyword provided by SecretEPDB. After selecting the “Submit” button, the corresponding search results will be displayed at the result webpage. Users can click a database ID to visualize the detailed information of the current entry. Note that if a protein entry was originally extracted from the UniProt database, then a link pointing to the corresponding UniProt webpage is also provided at the search result webpage. Users click the UniProt ID to transfer to the corresponding UniProt webpage of this entry.

**Figure 6:**
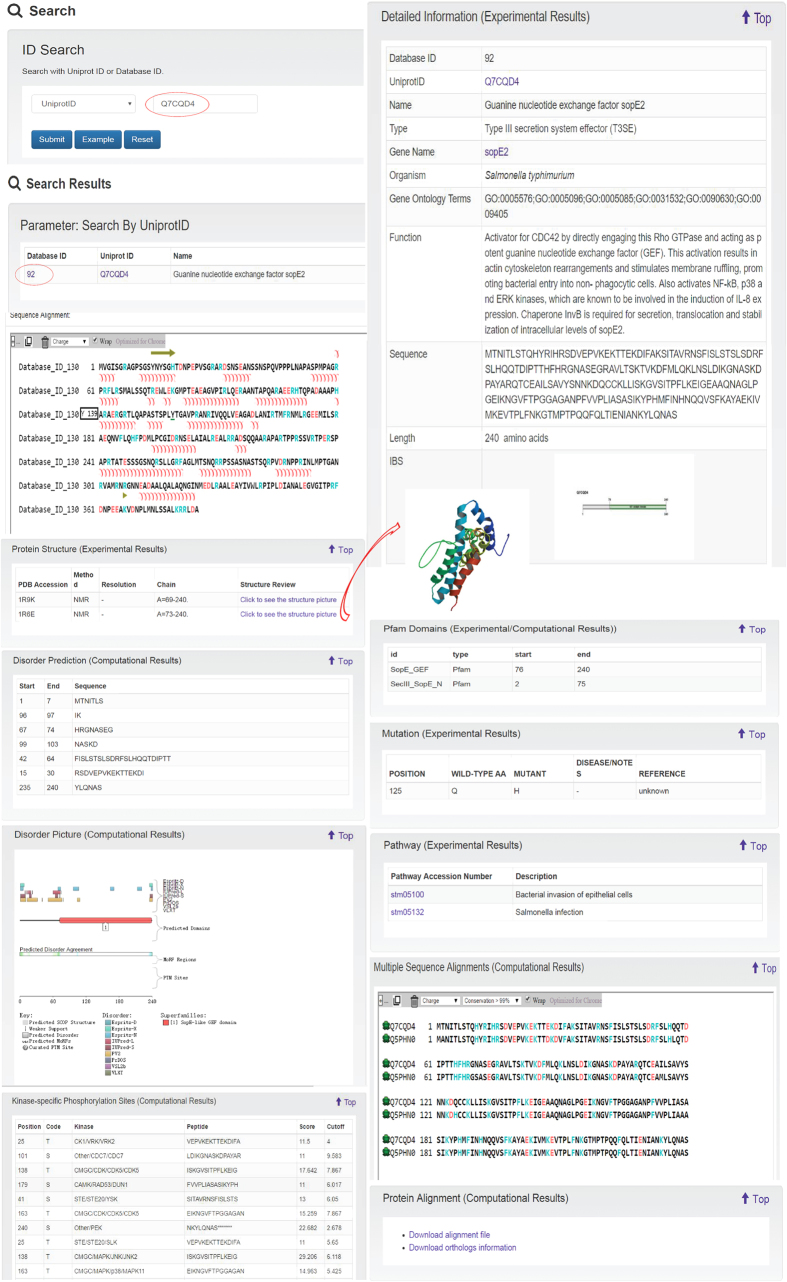
Output of the sample search against SecretEPDB using UniProt ID “Q7CQD4” as the query. The results are displayed and organized by different annotation categories, including protein detailed information, sequence alignment, protein structure, multiple sequence alignments, Pfam domain, disorder region prediction, disorder picture, protein mutation and metabolic/signaling pathway.

By way of example, binding of *Salmonella* to the surface of intestinal epithelial cells activates a T3SS that secretes several T3SEs including SopE, SopE2 and SopB: these effectors mimic host cell proteins and thereby activate host cell regulatory proteins to initiate actin cytoskeleton rearrangements. For SopE2, first discovered in more than ten years ago^62^,^63^,^64^, this occurs because it mimics host cell guanine nucleotide exchange factors, or GEFs. Users with an interest in this biological phenomena who are investigating the UniProt ID “Q7CQD4”, corresponding to SopE2will access results that are displayed and organized according to the major annotation categories (Fig. 6).

In order to simplify entry browsing, each entry can be displayed in accordance with their type (i.e. T3SE, T4SE or T6SE) at the browse webpage. Users can download the entire database of SecretEPDB in the SQL format. Alternatively, protein structures and MSAs of the entries are available for download. To collect the up-to-date experimental findings of secretion effectors, we provide an option for researchers to submit their recent results to SecretEPDB via an online entry submission webpage (available at http://secretepdb.erc.monash.edu.au/submission.jsp). At the “Submission” webpage, two submission modules (quick submission and formal submission) are available for users to submit their recently discovered effectors to SecretEPDB. When using the ‘quick submission’ module, users can simply submit a new effector protein by providing brief information, such as subject (describing the protein name or identity) and description (providing the UniProt accession/link, PubMed ID/link or the title of the literature paper if possible). After successfully receiving the request, the database administrator will then carefully review the submission and accordingly update the database after verification. When using the ‘formal submission’ module, users are required to provide more detailed information necessary for annotating the entries that they would like to submit. Such information includes contact information, and protein general information, including protein name, species, gene name, molecular weight, effector type, protein sequence, etc. Users are also encouraged to provide additional (optional) information such as Uniprot ID, protein structural and functional annotations. Each submission will be subject to further scrutiny prior to being included in the database and made publicly available. Furthermore, our database team will regularly maintain and update the database by means of searching recently published literature papers and keeping track of the updates of UniProt and PubMed.

We have also made available a ‘Timeline’ module (http://secretepdb.erc.monash.edu.au/timeline.action), through which users can readily view the information of each major recent update. This enables users to rapidly track recent update history, time and entries included in all recent major updates.

## Conclusion

In this work, we develop a new web-based knowledgebase, termed SecretEPDB, which provides comprehensive annotations of effector proteins of three major bacterial secretion systems T3SS, T4SS and T6SS. The annotations provided by SecretEPDB include protein functional annotation, protein 3D structure, Pfam domains, metabolic pathways, and protein evolutionary information. All entries documented in SecretEPDB have been manually annotated and experimentally verified. We anticipate that SecretEPDB will be a useful resource for generating novel hypothesis of the translocation mechanisms and function of secretion effectors and contribute to a better understanding of the functional characterization of these proteins and their corresponding secretion systems.

## Additional Information

**How to cite this article**: An, Y. *et al*. SecretEPDB: a comprehensive web-based resource for secreted effector proteins of the bacterial types III, IV and VI secretion systems. *Sci. Rep.* **7**, 41031; doi: 10.1038/srep41031 (2017).

**Publisher's note:** Springer Nature remains neutral with regard to jurisdictional claims in published maps and institutional affiliations.

## Supplementary Material

Supplementary Information

## Figures and Tables

**Table 1:** Statistical summary of the three types of effector proteins collected from the literature.

Type	Reference	Number of entries in the reference	Number of entries included in SecretEPDB
T3SE	Dong, X. *et al*.^21^; Wang, Y. *et al*.^28^	150	150
	Arnold, R. *et al*.^20^	100	94
	Tay, D. M. *et al*.^41^	504	334
	Yang, X. *et al*.^14^	283	260
	Dong, X. *et al*.^32^	1215	704
T4SE	Bi, D. *et al*.^31^	239	239
	Zou, L. *et al*.^29^	340	340
	Wang, Y. *et al*.^42^	347	347
	Lifshitz, Z. *et al*.^43^	290	290
T6SE	Li, J. *et al*.^33^	107	88
	Salomon, D.* et al.*^24^	6	6
	Russell, A. *et al*.^44^	50	40
	Russell, A. *et al*.^45^	61	38
