# Supplementary material for: SecretEPDB: a comprehensive web-based resource for secreted effector proteins of the bacterial types III, IV and VI secretion systems
Source: Sci Rep. 2017 Jan 23;7:41031. doi: 10.1038/srep41031 (PMC5253721; doi:10.1038/srep41031)
Supplement: Supplementary Information [file srep41031-s1.pdf]

SecretEPDB: a comprehensive web-based resource for secreted effector proteins of the bacterial types III, IV and VI secretion systems

Yi An[1,2,†], Jiawei Wang[3,†], Chen Li[4], Jerico Revote[5], Yang Zhang[1,*], Thomas Naderer[6], Morihiro Hayashida[7], Tatsuya Akutsu[7], Geoffrey I. Webb[2,*], Trevor Lithgow[4] and Jiangning Song[2,6,*]

[1]College of Information Engineering, Northwest A&F University, Yangling 712100, China, [2]Monash Centre for Data Science, Faculty of Information Technology, Monash University, Melbourne, VIC 3800, Australia, [3]School of Electronic and Computer Engineering, Peking University, Beijing 100871, China, [4]Infection and Immunity Program, Biomedicine Discovery Institute and Department of Microbiology, Monash University, Melbourne, VIC 3800, Australia, [5]Monash Bioinformatics Platform, Monash University, Melbourne, VIC 3800, Australia, [6]Infection and Immunity Program, Biomedicine Discovery Institute and Department of Biochemistry and Molecular Biology, Monash University, Melbourne, VIC 3800, Australia, [7]Bioinformatics Center, Institute for Chemical Research, Kyoto University, Uji, Kyoto 611-0011, Japan

*To whom correspondence should be addressed: zhangyang@nwsuaf.edu.cn; Geoff.Webb@monash.edu; Jiangning.Song@monash.edu.

[†]These two authors contributed equally to this work.

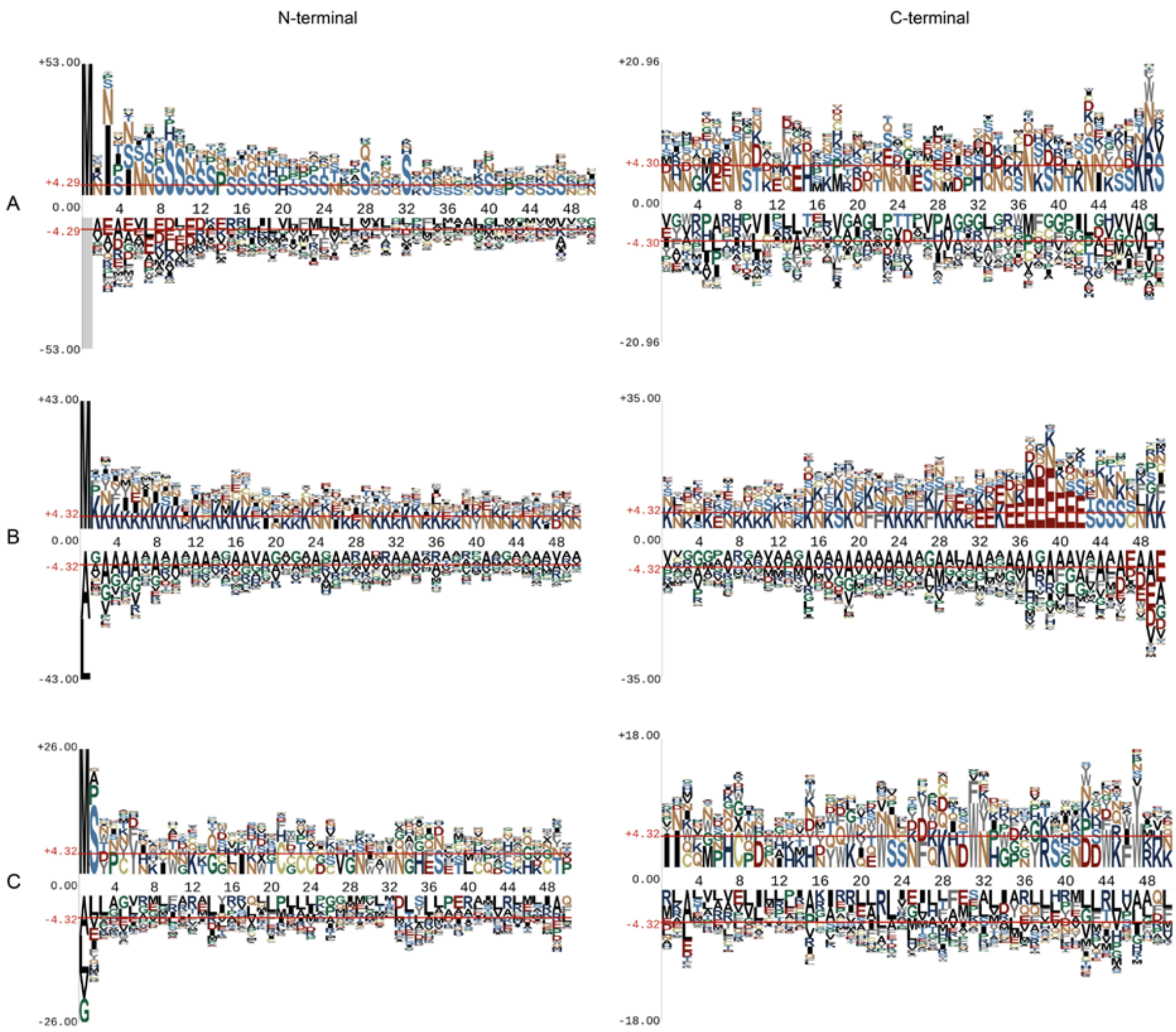

Supplementary Figure S1. Sequence logo representations showing the amino acid conservation and preferences of N- and C-terminal motifs of (A) T3SEs, (B) T4SEs, and (C) T6SEs. In all panels the x-axis represents residue position: amino acids above the x-axis are favored, while those below the x-axis are disfavored at the corresponding positions. Note that these sequence logos are generated using the whole set of each type of secreted effectors, rather than species-specific subsets of sequence data.
